# Echocardiographic Evaluation of Systolic and Diastolic Cardiac Functions in Pediatric Cirrhosis: Unmasking Subclinical Alterations and Correlation with Liver Severity Scores

**DOI:** 10.3390/children13091217

**Published:** 2026-09-08

**Authors:** Mehmet Öncül, Şükrü Güngör, Özlem Elkıran, Fatma İlknur Varol, Emre Gök, Fatih Tat, Hanım Bayram, Selçuk Erdoğan, Elif Damar Güngör, Yurday Öncül

**Affiliations:** 1 Department of Pediatric Cardiology, Faculty of Medicine, İnonu University, 44280 Malatya, Türkiye; ozlem.elkiran@inonu.edu.tr (Ö.E.);; 2 Department of Pediatric Gastroenterology, Faculty of Medicine, İnonu University, 44280 Malatya, Türkiye; sukru.gungor@inonu.edu.tr (Ş.G.); ilknur.varol@inonu.edu.tr (F.İ.V.); emre.gok@inonu.edu.tr (E.G.);; 3 Department of Ophthalmology Clinic, Faculty of Medicine, İnonu University, 44280 Malatya, Türkiye; 4 Department of Pediatric Hematology, Faculty of Medicine, İnonu University, 44280 Malatya, Türkiye; yurday.oncul@inonu.edu.tr

**Keywords:** liver failure, heart, echocardiogram, children, cirrhosis

## Abstract

**Highlights:**

**What are the main findings?**

- Subclinical Myocardial Dysfunction: Although resting systolic functions in pediatric cirrhosis patients appear preserved due to hyperdynamic circulation, tissue Doppler imaging reveals a concomitant latent global myocardial dysfunction and diastolic relaxation impairment.
- Hepatocardiac Axis and Hemodynamic Load: Elevated routine liver scores, such as PELD, FIB-4, and APRI, are directly associated with the filling pressures and hemodynamic overload of both the right and left ventricles of the heart.

**What are the implications of the main findings?**

- Diastolic Dysfunction Parameters: In patients with normal left ventricular systolic functions, the Mitral E velocity measured by PW Doppler is increased, whereas the Mitral E/A ratio and the tissue-level mitral e′/a′ ratio—which reflect early-stage relaxation impairment—are significantly lower in the cirrhosis group. Among the diastolic time intervals, Mitral DT, IVRT, and IVCT durations are significantly prolonged in the pediatric cirrhosis group.
- Clinical Correlation Success: Non-invasive clinical tools used for monitoring pediatric cirrhotic patients, such as PELD, FIB-4, and APRI, demonstrate significant clinical correlation success in detecting subclinical involvements in the liver-heart axis at an early stage. In parallel with the increase in the PELD score, indicators of hyperdynamic circulation, such as heart rate (HR) and ejection fraction (EF) values, increase; conversely, Mitral E/A and e′/a′ ratios, which reflect relaxation impairment, exhibit a significant negative correlation.

**Abstract:**

Objective: This prospective study evaluated systolic and diastolic cardiac functions in pediatric cirrhosis using conventional and tissue Doppler imaging (TDI), and explored their correlation with liver severity scores. Methods: Conducted at Inonu University, we enrolled 75 pediatric cirrhotic patients (44 compensated, 31 decompensated) from Pediatric Gastroenterology and 61 controls of similar age and sex without structural/functional heart disease from Pediatric Cardiology outpatients. All underwent clinical, laboratory, and echocardiographic assessments. To address multiple testing, Bonferroni correction was applied. Adjustments for age, sex, and disease severity were integrated into the correlation matrices to control for confounding parameters. Results: The cirrhosis cohort exhibited significantly higher heart rates, ejection fractions, and aortic/pulmonary velocity-time integrals than controls (*p* < 0.05). Conversely, growth-independent diastolic indices revealed distinct relaxation impairment: transmitral E/A and annular e′/a′ ratios were significantly lower, while Mitral E/e′ was elevated (*p* < 0.05). Deceleration time, isovolumetric relaxation time, and Myocardial Performance Index (MPI) were significantly prolonged (*p* < 0.01). Decompensated patients demonstrated significantly higher left ventricular filling pressures (Mitral E/e′) than compensated patients and controls (*p* = 0.002). After adjusting for age and sex, the Pediatric End-Stage Liver Disease (PELD) score maintained significant positive correlations with heart rate (*r* = 0.408) and Mitral E/e′ (*r* = 0.433) (*p* ≤ 0.002). FIB-4 and APRI scores showed positive correlations with VTI and TAPSE, markers of right ventricular volume overload (*p* < 0.05). Conclusions: Hyperdynamic circulation appears to mask resting systolic metrics in pediatric cirrhosis. However, prolonged diastolic phases and increased MPI suggest presence of subclinical myocardial dysfunction, especially during clinical decompensation. Routine non-invasive liver staging scores show potential clinical utility as ancillary screening tools for early cardiovascular risk evaluation.

## 1. Introduction

Cirrhotic cardiomyopathy (CCM) is a clinical condition characterized by an impaired myocardial contractile response to stress, diastolic dysfunction, and electrophysiological abnormalities in individuals with chronic liver disease, despite normal or increased cardiac output at rest and in the absence of primary heart disease [1,2,3,4].

Chronic liver disease and cirrhosis represent a complex pathological state that is not confined to the liver parenchyma but instead exhibits multisystemic involvement. One of the most characteristic features in the pathophysiology of cirrhosis is the “Hyperdynamic Circulation Syndrome,” which develops secondary to splanchnic and systemic vasodilation, leading to a reduction in effective arterial blood volume [5,6]. To compensate for decreased systemic vascular resistance and arterial hypotension, the body chronically activates the sympathetic nervous system and the renin–angiotensin–aldosterone system. This leads to the release of vasodilators from the liver into the circulation, the release of factors that suppress cardiac function, and arterial vasodilation [6].

These chronic hemodynamic changes that develop during the course of cirrhosis trigger structural and functional changes in myocardial tissue over time. This clinical condition, defined in the literature as CCM, occurs in cirrhotic patients with no known primary heart disease as a consequence of chronic hyperdynamic circulation, myocardial fibrosis, and defects in calcium reuptake at the cellular level [7].

Cardiac dysfunction in cirrhosis usually remains undetected and is widely overlooked. This is because findings remain asymptomatic at rest; however, under physiological or pharmacological stress, it can transform into overt heart failure, leading to significant morbidity and mortality [8]. Cardiovascular dysfunction refers to any abnormality or impairment in the function of the cardiovascular system, which comprises the heart, blood vessels, and blood [9,10]. Traditionally, CCM is characterized by a decrease in left ventricular relaxation capacity—namely diastolic dysfunction—and electrophysiological anomalies, contrasting with normal or increased systolic functions at rest [11,12].

Although cardiac involvement and diagnostic criteria have been extensively studied in adult cirrhotic patients, the liver-heart axis in pediatric and young age groups remains an insufficiently elucidated topic [13]. The fact that myocardial tissue in children possesses higher elasticity and a more robust compensatory reserve compared to adults makes it difficult for conventional echocardiographic cut-off values to capture subclinical cardiac damage, thereby leading to diagnostic paradoxes where resting systolic metrics like ejection fraction (EF) remain falsely reassuring. Consequently, there is a critical knowledge gap in pediatric hepatology regarding which specific parameters can unmask occult cirrhotic cardiomyopathy (CCM) before clinical decompensation occurs. To address this, the evaluation of active left ventricular relaxation phases [isovolumetric relaxation time (IVRT), isovolumetric contraction time (IVCT), and deceleration time (DT)] and the holistic evaluation of global contractile indices [myocardial performance index (MPI)] provide incremental diagnostic value over standard imaging unmasking latent myocardial alterations independently of standard hyperkinetic resting indices [14].

Conventional Doppler echocardiography has limitations and rarely differentiates normal from pseudo normal filling patterns. Tissue Doppler Imaging (TDI) offers a distinct advantage as a highly sensitive methods for evaluating left ventricular filling dynamics independently of preload alterations [15].

In recent years, the Pediatric End-Stage Liver Disease (PELD) score and biochemical indices such as FIB-4 and APRI have been widely utilized in children to reflect the severity of liver disease and parenchymal fibrosis burden non-invasively [16]. However, the linear relationship of these non-invasive clinical and laboratory scores with right and left ventricular filling dynamics, velocity-time integral (VTI), and longitudinal right ventricular functions, as well as their efficacy in predicting subclinical cardiovascular load, has not been sufficiently investigated in the pediatric literature.

According to the contemporary criteria proposed by the Cirrhotic Cardiomyopathy Consortium (1), a definitive diagnosis of cirrhotic cardiomyopathy (CCM) requires advanced assessments including global longitudinal strain (GLS), electrophysiological evaluations, and stress-induced contractile reserve testing. Given the logistical and physiological constraints in the pediatric population, evaluating the complete clinical phenotype remains highly challenging. Nevertheless, the assessment of active left ventricular relaxation phases, such as isovolumetric relaxation time (IVRT), isovolumetric contraction time (IVCT), deceleration time (DT), and the holistic evaluation of global contractile indices like the myocardial performance index (MPI), provide incremental diagnostic value over standard imaging. Delineating these specific parameters is critical to unmasking occult cardiac involvement before overt clinical decompensation occurs.

To address these gaps, this prospective study was designed with clearly defined endpoints to evaluate the subclinical liver–heart axis alterations in children. The primary objective was to determine the presence and severity of subclinical global myocardial dysfunction in a pediatric cirrhotic cohort compared to healthy controls, with a specific focus on tissue Doppler imaging (TDI)-derived filling pressures (E/e′) and global performance indicators (MPI, IVRT, IVCT, DT). The secondary objective was to evaluate whether routinely used non-invasive hepatic fibrosis indices (FIB-4, APRI, AAR) and clinical mortality scores (PELD, MELD) correlate with and effectively reflect this subclinical cardiovascular burden, thereby serving as early ancillary screening tools in the clinical follow-up of pediatric cirrhosis.

## 2. Method

### 2.1. Study Design

The study was conducted among patients who applied between August 2025 and February 2026. It included patients aged 0–18 years diagnosed with chronic liver failure and cirrhosis and followed up at the Department of Pediatric Gastroenterology, Inonu University Faculty of Medicine Research Hospital, and healthy children who presented to the Pediatric Cardiology outpatient clinic with complaints of heart murmur or non-cardiac chest pain, and in whom no cardiological or systemic pathology was detected via physical examination and echocardiography. The study included a total of 75 patients with confirmed liver cirrhosis, encompassing compensated and decompensated cases of end-stage chronic liver failure, and 61 age/sex-matched cardiac evaluation controls without identified structural or functional heart disease.

### 2.2. Inclusion Criteria

Patient Group Selection: All patients aged 0–18 years followed for chronic liver failure with a confirmed diagnosis of chronic liver disease or cirrhosis based on clinical, laboratory, radiological, and/or histopathological findings were included.

Healthy Control Group: Healthy volunteers of similar age and gender, with no known chronic, systemic, or cardiac diseases, were included.

### 2.3. Exclusion Criteria

A history of known congenital or acquired heart disease, or arrhythmia.

Co-existing chronic conditions that could directly affect cardiac function, such as primary pulmonary hypertension, chronic or dialysis-dependent renal failure, or diabetes mellitus.

Suboptimal echocardiographic image quality precluding accurate measurements.

### 2.4. Diagnostic Criteria for Liver Cirrhosis [17]

#### Compensated Cirrhosis

The diagnosis of compensated cirrhosis requires meeting at least one of the following three criteria:

Histological Evidence: Pathological proof of cirrhosis via liver biopsy.

Endoscopic Features: Endoscopic evidence of portal hypertension—such as esophageal varices, gastric fundal varices, or portal hypertensive gastropathy—after excluding non-cirrhotic causes of portal hypertension.

Imaging Findings: Diagnostic features of cirrhosis or portal hypertension observed on ultrasound, computed tomography, or magnetic resonance imaging.

### 2.5. Decompensated Cirrhosis

The diagnosis of decompensated cirrhosis requires a baseline diagnosis of cirrhosis, accompanied by: Complications of portal hypertension (e.g., ascites, spontaneous bacterial peritonitis, esophageal variceal bleeding), and/or Impaired liver function (synthetic/metabolic dysfunction).

### 2.6. Histopathology Grading & Staging [18]

Grading (Inflammatory Activity: Grades 0–3): Ranges from Grade 0 (no inflammation or fibrosis) to Grade 3 (severe inflammation accompanied by ballooning degeneration and necrosis, without fibrosis).

Staging (Fibrosis/Cirrhosis: Stage 4): Disease progression culminates in Stage 4 (Cirrhosis), which is characterized by nodular regeneration, bridging fibrosis, and distortion of the normal hepatic architecture.

### 2.7. Scoring Calculations

The following fibrosis scoring indices were calculated:

FIB-4 Score: FIB-4 = (Age × AST)/(Platelet Count × √ALT) [19].

APRI Score (AST to Platelet Ratio Index): APRI = ((AST/ULN AST) ÷ Platelet Count) × 100 (AST upper limit of normal [ULN] was standardized to 35 U/L for this study) [20].

AST/ALT Ratio (De Ritis Ratio): AST/ALT Ratio = AST/ALT [21].

### 2.8. Mortality Score Calculator

MELD Na: In children aged 12 years and older, scores were calculated by incorporating data on dialysis requirement, serum creatinine, total bilirubin, INR, and serum sodium (Na) [22].

PELD: In children under 12 years of age, evaluations were performed by recording data on age, albumin, bilirubin, INR, and the presence of growth failure [23].

### 2.9. Echocardiographic Evaluation

All patients underwent echocardiographic examinations performed by an expert pediatric cardiologist who was fully blinded to the clinical laboratory data, mortality scores, and fibrosis indices of the participants using a General Electric (GE Vivid E9) ultrasound system equipped with appropriate transducers. Detailed evaluations were conducted in the left lateral decubitus position in accordance with the guidelines of the American Society of Echocardiography. Imaging modalities included M-mode, two-dimensional (2D) echocardiography, PW Doppler, continuous-wave (CW) Doppler, color Doppler, and TDI. Images obtained from standard parasternal and apical windows were analyzed. Left ventricular EF was derived via M-mode echocardiography in the parasternal long-axis view. Doppler recordings of diastolic mitral inflow were obtained from the apical four-chamber view, and measurements were recorded as the average of two consecutive cardiac cycles. Peak early diastolic (E) and late diastolic (A) filling velocities were measured across both the mitral and tricuspid valves to calculate the E/A ratios.

For tissue Doppler measurements, all interval parameters were obtained within a single cardiac cycle. TDI data were acquired from the apical four-chamber view. The Doppler index was determined by averaging three distinct measurements for each tissue site. Peak early diastolic annular velocity (e′) and late diastolic annular velocity (a′) were measured at the septal mitral annulus using TDI. In addition, IVRT, IVCT, ET, and DT were measured, and the E/e′ ratio was calculated. The myocardial performance index (MPI) was calculated using the formula “(IVCT + IVRT)/ET”. Right ventricular fractional area change (FAC) was calculated by measuring the right ventricular end-systolic area (ESA) and end-diastolic area (EDA) using the 2D area tracking’s method. During contrast echocardiography, intrapulmonary shunting- indicative of hepatopulmonary syndrome and intrapulmonary vascular dilations- was evaluated to assess its role in driving hyperdynamic changes. This was based on the appearance of microbubbles in the left cardiac chambers within 3–6 cardiac cycles following the intravenous injection of an echocardiographic contrast agent (1.5% agitated saline).

### 2.10. Power Analysis

Given the absence of a direct pediatric reference study examining tissue Doppler findings alongside clinical prognostic scores in cirrhosis, the sample size estimation was calculated based on a moderate effect size (d = 0.50) for our primary predefined echocardiographic endpoints: the Mitral E/e′ ratio (reflecting left ventricular filling pressure) and TAPSE (reflecting right ventricular longitudinal function). Assuming a Type I error rate (α) of 5% (0.05) and a targeted statistical power (1 − β) of 0.85, a minimum of 39 subjects per group was required. Our final enrollment of 136 subjects (75 in the cirrhosis group and 61 in the control group) exceeded this minimum threshold, ensuring adequate statistical power to detect meaningful differences in these primary cardiac outcomes.

### 2.11. Statistical Analysis

Statistical Package for Social Sciences (SPSS) for Windows 22.0 (SPSS, Inc., Chicago, IL, USA) program was used to assess the data statistically. The conformity of variables to a normal distribution was assessed using both visual and analytical methods (Kolmogorov–Smirnov and Shapiro–Wilk tests). Descriptive statistics were expressed as mean ± standard deviation for normally distributed continuous variables, and as median (interquartile range) for non-normally distributed continuous variables. Categorical variables were presented as frequencies and percentages (%).

For the comparison of continuous variables between the cirrhosis and control groups, Student’s *t*-test was used for normally distributed parameters, whereas the Mann–Whitney *U* test was employed for non-normally distributed parameters. Statistical differences in echocardiographic parameters among the healthy control group, compensated cirrhosis group, and decompensated cirrhosis group were evaluated using One-Way Analysis of Variance (ANOVA). Categorical data were compared using the Chi-square test or Fisher’s exact test. The relationships between echocardiographic parameters and PELD/MELD scores, as well as fibrosis indices (FIB-4, APRI, AAR), were evaluated using Pearson or Spearman correlation analysis. To account for the multiple testing problem and minimize the risk of Type I errors in the correlation matrices, a Bonferroni multiplicity correction was calculated. A strict global alpha threshold for absolute statistical significance (0.05/80 = 0.000625) was defined, considering 16 cardiovascular parameters and 5 different liver prognostic indices. Additionally, an exploratory family-wise correction per hepatic scoring system (0.05/16 = 0.003125) was provided to retain clinical visibility for robust primary endpoints (such as PELD and filling dynamics).

The optimal cut-off thresholds for echocardiographic and hemodynamic parameters in predicting decompensated cirrhosis were primarily determined using the Receiver Operating Characteristic (ROC) curve analysis based on the Youden Index (Sensitivity + Specificity − 1) to maximize structural diagnostic accuracy. For clinically critical screening parameters—specifically the Mitral E/e′ ratio—the optimal cut-off was purposely selected to ensure 100% sensitivity and 100% negative predictive value (NPV), prioritizing the absolute avoidance of false negatives in identifying subclinical decompensation phases. Exact 95% Confidence Intervals (CIs) were calculated for each Area Under the Curve (AUC) to indicate parameter precision. For all statistical analyses, a *p*-value of <0.05 was considered statistically significant.

## 3. Results

### 3.1. Demographic, Anthropometric, and Laboratory Findings

A total of 136 subjects were enrolled in the study, comprising 75 (55.1%) patients with cirrhosis and 61 (44.9%) healthy controls. The mean age of the cohort was 7.18 ± 5.67 years, and 60.1% (*n* = 82) of the participants were male.

In the anthropometric assessment, the cirrhosis group exhibited significantly lower weight Z-scores (−1.19 ± 1.69) and height Z-scores (−1.32 ± 1.82) compared to the control group (*p* < 0.001). Additionally, the prevalence of malnutrition was significantly higher in the cirrhosis group (*p* = 0.003).

When laboratory parameters were compared, erythrocyte indices (Hb, Htc), platelet parameters (PLT, PCT), as well as total protein and albumin levels, were found to be significantly lower in patients with cirrhosis than in the control group (*p* < 0.05). Conversely, RDW and PDW values, along with liver function tests (AST, ALT, GGT, ALP, and bilirubin fractions), were significantly higher in the cirrhosis group compared to the control group (*p* < 0.05). All demographic, anthropometric, and laboratory data are presented detail in Table 1.

### 3.2. Hemodynamic and Echocardiographic Findings

In the cirrhosis group, HR was significantly higher (*p* = 0.008), whereas peripheral oxygen saturation was significantly lower compared to the control group. Regarding structural left ventricular M-mode measurements, interventricular septal thickness at end-systole (IVSs) was significantly greater in the cirrhotic group than in the control group (*p* = 0.044), while no significant differences were observed in other M-mode parameters between the two groups.

Among the indicators of left ventricular systolic function, EF (*p* = 0.012) and fractional shortening (FS) (*p* = 0.009) were significantly higher in the cirrhosis group. Similarly, aortic VTI and pulmonary VTI values were markedly increased in the cirrhosis group compared to the control group (*p* < 0.001).

Evaluation of left ventricular diastolic inflow and tissue Doppler parameters revealed that while Mitral E was significantly higher in the cirrhosis group (*p* < 0.001), no significant difference was observed in Mitral A. Consequently, the transmitral filling velocity ratio (Mitral E/A) was significantly lower in the cirrhosis group than in the control group (*p* = 0.010).

On TDI measurements, peak late diastolic annular velocity (Mitral a′) was significantly higher in the cirrhosis group (*p* = 0.002), whereas peak early diastolic annular velocity (Mitral e′) showed no significant difference. However, the annular tissue mitral e′/a′ ratio was significantly lower in the cirrhosis group (*p* = 0.006). The Mitral E/e′ ratio, which is the most sensitive non-invasive indicator of left ventricular filling pressures, was significantly higher in the cirrhosis group compared to the control group (*p* = 0.001).

In terms of diastolic time intervals, mitral deceleration time (DT) (*p* = 0.001 IVRT (*p* < 0.001), and IVCT (*p* = 0.007) were significantly prolonged in the cirrhosis group. The myocardial performance index (MPI), which reflects overall left ventricular function and timing abnormalities, was also markedly higher (prolonged) in the cirrhosis group (*p* < 0.001).

Regarding right ventricular longitudinal function indicators, tricuspid annular plane systolic excursion (TAPSE) (*p* = 0.005) and FAC (*p* = 0.023), as well as right ventricular filling velocities including peak early diastolic tricuspid velocity (Tricuspid E) (*p* < 0.001) and peak late diastolic tricuspid velocity (Tricuspid A) (*p* < 0.001), were all significantly higher in the cirrhosis group. A comparative analysis of the participants’ hemodynamic and echocardiographic parameters is presented in Table 2.

### 3.3. Echocardiographic Changes According to Cirrhosis Stage (Compensated vs. Decompensated)

When patients with cirrhosis were stratified into compensated (*n* = 44) and decompensated (*n* = 31) subgroups and compared with the healthy control group, hemodynamic data revealed that HR was significantly higher in the decompensated group than in the control group (*p* = 0.027), whereas it was similar between the compensated and control groups. Conversely, peripheral oxygen saturation was significantly lower in both the compensated and decompensated cirrhosis groups compared to the control group (*p* < 0.001).

Regarding left ventricular systolic function parameters EF (*p* = 0.037) and FS (*p* = 0.034), as well as stroke volume indices including aortic VTI (*p* < 0.001) and pulmonary VTI (*p* < 0.001), were significantly higher in both the compensated and decompensated cirrhosis groups than in the control group.

Evaluation of diastolic and tissue Doppler parameters showed that Mitral E, Mitral a′, mitral DT, IVRT, IVCT, and MPI values were significantly higher or prolonged in both the compensated and decompensated cirrhosis groups compared to the control group (*p* < 0.05). In contrast, the Mitral E/A and annular tissue mitral e′/a′ ratios were significantly lower compared to the control group only in patients with compensated cirrhosis. Conversely, the Mitral E/e′ ratio was significantly higher only in the decompensated cirrhosis group compared to the control group (*p* = 0.002).

Among the right ventricular parameters, TAPSE was significantly higher exclusively in the decompensated cirrhosis group (*p* = 0.005). However, Tricuspid E (*p* < 0.001) Tricuspid A (*p* = 0.001) were significantly higher in both the compensated and decompensated groups than in the healthy control group. All cardiac data classified according to compensation status are presented in Table 3.

The PELD score demonstrated significant positive correlations with hyperdynamic circulation indicators, including HR and EF, as well as right ventricular filling velocities (Tricuspid E and Tricuspid A) and the Mitral E/e′ ratio, which serves as a surrogate marker for left ventricular filling pressures. Conversely, significant negative correlations were observed between the PELD score and metrics reflecting left ventricular relaxation impairment, namely the transmitral Mitral E/A ratio and the annular tissue mitral e′/a′ ratio. The adult-oriented MELD score exhibited a strong positive correlation exclusively with peak Tricuspid A, showing no statistically significant relationships with other cardiovascular parameters.

In terms of non-invasive indices reflecting hepatic fibrosis burden, the FIB-4 score demonstrated highly significant positive correlations with TAPSE, as well as both aortic and pulmonary VTI. In parallel with increasing APRI scores, linear increases were observed in HR, EF, FS, and markers of right ventricular hemodynamic load (Pulmonary VTI and Tricuspid E), whereas peripheral oxygen saturation decreased significantly. Finally, the AST/ALT ratio (AAR) showed significant positive correlations with HR and the Mitral E/e′ ratio, while displaying a significant negative relationship with TAPSE values. The results of the Pearson correlation analysis evaluating the linear relationships between echocardiographic parameters, liver disease severity, and non-invasive scores of parenchymal fibrosis burden are presented in Table 4.

### 3.4. Diagnostic Performance of Cardiac Parameters in Predicting Decompensated Cirrhosis (ROC Curve Analysis)

To determine the diagnostic performance and optimal cut-off values of echocardiographic and hemodynamic parameters in predicting the development of decompensated cirrhosis, a Receiver Operating Characteristic (ROC) curve analysis was performed using the Youden Index maximization method. Following the protocol for multiplicity control, the statistical significance of the Areas Under the Curve (AUC) was evaluated against strict family-wise adjusted significance thresholds. The analysis revealed that Mitral E exhibited the highest area under the curve value for predicting decompensated cirrhosis (AUC: 0.758, 95% CI: 0.677–0.827, *p* < 0.001). Additionally, pulmonary VTI, which serves as an indirect surrogate marker of right ventricular stroke volume, was identified as another significant predictor for decompensation (AUC: 0.727, 95% CI: 0.644–0.800, *p* < 0.001).

A cut-off value of >6.14 for the Mitral E/e′ ratio—reflecting left ventricular filling pressures—provided a sensitivity of 100.00% and a negative predictive value (NPV) of 100.00% in classifying patients with decompensated cirrhosis (AUC: 0.654, 95% CI: 0.568–0.734, *p* = 0.009, specificity: 29.25%). Furthermore, after multi-comparison adjustment, aortic VTI (AUC: 0.679, 95% CI: 0.594–0.757, *p* = 0.002), Tricuspid E (AUC: 0.691, 95% CI: 0.606–0.768, *p* = 0.001), and Tricuspid A (AUC: 0.669, 95% CI: 0.583–0.747, *p* = 0.004) all demonstrated statistically significant predictive capacities for identifying clinical decompensation phases. The optimal cut-off values, sensitivities, specificities, positive predictive values (PPV), and NPVs for all analyzed parameters are presented in detail in Table 5.

## 4. Discussion

This study contributes to the limited literature investigating the development of subclinical cirrhotic cardiomyopathy in pediatric patients and evaluating the predictive value of non-invasive prognostic scores reflecting liver disease severity, using both conventional and tissue Doppler echocardiography in comparison with a healthy control cohort. Our findings demonstrate that although resting systolic function appears to be preserved or even elevated secondary to a hyperdynamic circulation, latent global myocardial dysfunction and relaxation impairment at the tissue level distinctly accompany the clinical presentation in the pediatric cirrhotic population.

Considering that data regarding the pediatric age group remain limited in the literature, a notable finding of our study is that routine clinical scores, such as PELD, FIB-4, and APRI, reliably reflect not only hepatic fibrosis but also right and left ventricular filling dynamics and increased diastolic load.

In our study, we observed that left ventricular systolic function markers, namely EF and FS, remained within normal limits but were statistically significantly higher in children with cirrhosis compared to the healthy control group. This phenomenon is directly associated with ‘Hyperdynamic Circulation Syndrome′—driven by splanchnic and systemic vasodilation, which is the most characteristic hemodynamic manifestation of liver cirrhosis—and the resulting chronic sympathetic compensation. It has been suggested in the literature that ejection fraction remains preserved in most cirrhotic patients until the end-stage of the disease, and that the heart may even exhibit a hyperkinetic response to compensate for reduced vascular resistance [12,24]. Indeed, the significantly higher heart rate observed in our patient cohort further supports this chronic sympathetic activation and hyperdynamic state. Another notable finding of our study is the distinct increase in both aortic and pulmonary VTI values in the cirrhosis group compared to the control group. Although no direct comparative study investigating aortic and pulmonary VTI values as reflections of stroke volume in pediatric cirrhosis cases was identified in the literature, we consider this increase to be a cardiovascular manifestation of the hyperdynamic circulation that develops secondary to chronic liver disease.

In cirrhotic cardiomyopathy, diastolic dysfunction is typically the earliest cardiac manifestation, usually preceding systolic dysfunction and remaining subclinical at rest. In our study, despite an increased peak early diastolic mitral inflow velocity measured by pulsed-wave Doppler, we found that the transmitral Mitral E/A ratio and the annular tissue mitral e′/a′ ratio was significantly lower in the cirrhosis group. Concurrently, among the diastolic time intervals, mitral DT, IVRT, and IVCT were significantly prolonged in the cirrhosis group. These findings support prior studies indicating that left ventricular compliance and relaxation capacity are impaired due to myocardial fibrosis and cellular-level calcium reuptake defects [25,26].

These findings are consistent with the data of Çelik et al. and Fattouh et al., who detected a decrease in the E/A ratio, prolongation of IVRT, and prolongation of Mitral DT time, which are considered indicators of diastolic dysfunction in children with cirrhosis [27,28]. Furthermore, the significantly higher Mitral E/e′ ratio observed in our patient cohort, which serves as an indicator of left ventricular filling pressures, is consistent with the subclinical presence of this relaxation impairment. Likewise, the elevated (prolonged) MPI value in the cirrhosis group compared to the control group, which provides an integrated assessment of both contraction and relaxation cycles [27,29], reveals that a latent global myocardial dysfunction underlies the seemingly hyperkinetic systolic functions, consequently leading to a shortened ET.

In addition to left-sided cardiac involvement, the manifestations of chronic hemodynamic and volume overload on the right heart during the course of cirrhosis are of critical importance for clinical management. In our study, we observed that right ventricular longitudinal function indicators, namely TAPSE and FAC, as well as right ventricular filling velocities, were all statistically significantly higher in the cirrhosis group compared to the control group. Similarly, López-Candales et al. (2014), in their study evaluating right ventricular function in adult patients with end-stage liver failure awaiting transplantation, reported that TAPSE and FAC values were markedly increased compared to the control group [30].

However, the finding that contraction indices (TAPSE, FAC) were elevated despite right ventricular end-systolic and end-diastolic areas (RVESA, RVEDA) remaining similar between the groups should not imply that the right heart function of these patients is entirely normal [31]. On the contrary, this finding confirms an exaggerated myocardial contractile response to the increased venous return and volume overload imposed on the right heart by the hyperdynamic circulation in chronic liver disease. Although the heart in cirrhotic cardiomyopathy appears to contract excessively at rest due to this hyperdynamic state [32], the cardiac reserve in these patients remains inadequate when they are exposed to stress, infection, surgery, or decompensation, which can rapidly precipitate overt heart failure.

The linear relationships observed between cardiac parameters and scoring systems reflecting liver disease severity and parenchymal fibrosis burden constitute one of the most novel findings of our study. In our pediatric cohort, in parallel with increasing PELD scores, indicators of a hyperdynamic circulation elevated significantly. Conversely, markers reflecting relaxation impairment, specifically the transmitral Mitral E/A and annular mitral e′/a′ ratios, exhibited significant negative correlations. Our most remarkable finding was the positive correlation between the PELD score and the Mitral E/e′ ratio (AUC: 0.654, 95% CI: 0.568–0.734, *p* = 0.009, specificity: 29.25%), which serves as the most sensitive non-invasive indicator of left ventricular filling pressures; this observation confirms that cardiac diastolic load increases as liver failure progresses. These data are consistent with recent literature reporting that higher PELD scores predict the development of CCM in children [33,34]. While the adult-oriented MELD score showed limited relationships with cardiovascular metrics due to age-specific cohort characteristics, the superior correlative success of the PELD score highlights its clinical utility in this population. Furthermore, the strong positive correlations demonstrated by the non-invasive fibrosis marker FIB-4 with pulmonary VTI evidence a compensatory hyperdynamic response developing in the right heart parallel to hepatic structural stiffening [16,35].

In light of all these data, it can be argued that non-invasive clinical tools such as PELD, FIB-4, and APRI, which are routinely used in the follow-up of pediatric cirrhotic patients, possess significant clinical correlative success in the early detection of subclinical involvement along the liver–heart axis [33,36]. Furthermore, our ROC curve analysis revealed that a cut-off value of >6.14 for the Mitral E/e′ ratio yielded 100% sensitivity and a 100% negative predictive value (NPV) in identifying cases of decompensated cirrhosis. While its modest AUC (0.654) and low specificity (29.25%) indicate that it should not be used as a standalone specific diagnostic metric, its clinical screening power remains outstanding for safely ruling out subclinical decompensation phases. Crucially, the validity of this filling pressure marker is further verified by our strict multi-comparison workflow; under the conservative family-wise Bonferroni correction, the direct correlation between the PELD score and the Mitral E/e′ ratio remained highly robust and statistically significant (*p* = 0.001, surviving the *p* < 0.003125 penalty). This dual strength suggests that the Mitral E/e′ ratio holds significant promise as a reliable ancillary screening criterion along the pediatric liver–heart axis.

### Study Limitations

Despite its strengths, several limitations of our study should be acknowledged. First, our research was conducted as a single-center study with a relatively small sample size, which is inherently related to the low incidence of cirrhosis in the pediatric age group and the rarity of pediatric CCM data. Second, cardiac functions were evaluated using conventional and tissue Doppler echocardiography at rest; however, advanced modalities such as speckle-tracking (strain) echocardiography or pharmacological/exercise stress testing, which could more sensitively unmask subclinical myocardial reserve insufficiency, were not performed. Finally, the simultaneous measurement of specific cardiac biomarkers, such as NT-proBNP or Troponin, which could provide biochemical evidence of cardiovascular involvement, was lacking in our cohort. However, despite these limitations, our multi-parameter Doppler approach provides significant clinical screening utility for detecting early subclinical cardiovascular load in children with chronic liver disease. We acknowledge that the significant differences in nutritional status (malnutrition Z-scores), anemia (Hb/Hct), and serum albumin levels between the cirrhosis and control groups could potentially exert confounding effects on our echocardiographic findings. Conditions such as chronic anemia and severe malnutrition are well-known triggers for increased sympathetic tone and peripheral vasodilation, which may independently exacerbate the hyperdynamic circulatory state observed in pediatric cirrhosis. Although our study primarily focuses on the primary hepatocardiac axis, these systemic variables may overlap with direct cirrhotic cardiomyopathy features. Future studies utilizing multivariate regression or covariance models are warranted to fully isolate the independent cardiac impact of metabolic and nutritional deficiencies from direct liver-induced myocardial remodeling.

## 5. Conclusions

In conclusion, this single-center, observational study with a limited sample size suggests that although resting systolic function appears to be preserved due to a hyperdynamic circulation, a latent global myocardial dysfunction and diastolic relaxation impairment at the tissue Doppler level distinctly accompany the clinical presentation in pediatric patients with cirrhosis. More importantly, elevated routine liver scores—such as PELD, FIB-4, and APRI—are directly associated with both right and left ventricular filling pressures, as well as the overall hemodynamic load of the heart. Specifically, while the high sensitivity and negative predictive value (NPV) offered by the echocardiographic Mitral E/e′ ratio provide potential utility in identifying subclinical alterations, its modest area under the curve (AUC: 0.654) and low specificity limit its independent diagnostic or prognostic power during the course of decompensated cirrhosis. These data suggest that routine liver staging scores can additionally serve as early ancillary indicators of hidden cardiovascular involvement in the follow-up of pediatric cirrhotic patients. Future multicenter and prospective studies incorporating external validation cohorts are required to better elucidate the long-term prognostic value of these non-invasive scores in the management of pediatric CCM.

## Figures and Tables

**Table 1:** Comparative Analysis of Demographic, Anthropometric, and Laboratory Findings Between the Groups.

Parameters	Control Group (*n* = 61) *n* (%)	Cirrhosis Group (*n* = 75) *n* (%)	*p*-Value
Gender (Female/Male)	27 (44.3)/34 (55.7)	27 (36.0)/48 (64.0)	0.327 *
Malnutrition (Absent/Present)	47 (77.0)/14 (23.0)	39 (52.0)/36 (48.0)	**0.003** *
* Mild Malnutrition	7 (11.5)	17 (22.7)	**0.008** *
* Moderate Malnutrition	3 (4.9)	6 (8.0)	**0.008** *
* Severe Malnutrition	1 (1.6)	10 (13.3)	**0.008** *
	**Mean ± SD**	**Mean ± SD**	
Age (years)	7.80 ± 5.51	6.68 ± 5.78	0.253 **
Weight Z-Score	−0.11 ± 1.38	−1.19 ± 1.69	**<0.001**
Height Z-Score	−0.08 ± 1.67	−1.32 ± 1.82	**<0.001**
BMI Z-Score	−0.03 ± 1.28	−0.47 ± 1.78	0.108
MUAC Z-Score	0.42 ± 1.10	−0.30 ± 1.42	0.050
WBC (10^3^ µL)	7.91 ± 3.10	8.30 ± 4.98	0.620
RBC (10^6^ µL)	6.14 ± 8.18	4.14 ± 0.96	0.141
Hemoglobin (g/dL)	12.67 ± 1.58	9.85 ± 1.91	**<0.001**
Hematocrit (%)	38.87 ± 4.79	31.56 ± 5.52	**<0.001**
Platelet (10^9^ L)	313.39 ± 77.31	188.32 ± 146.42	**<0.001**
PCT (%)	0.30 ± 0.06	0.23 ± 0.16	**0.017**
MCV (fL)	80.67 ± 4.21	78.08 ± 11.36	0.102
PDW (fL)	10.27 ± 1.68	11.65 ± 2.47	**0.008**
RDW (%)	39.13 ± 4.17	47.09 ± 16.13	**<0.001**
AST (U/L)	36.05 ± 12.25	241.40 ± 38.56	**<0.001**
ALT (U/L)	23.55 ± 12.70	161.04 ± 30.28	**<0.001**
Total Bilirubin (mg/dL)	0.43 ± 0.18	8.17 ± 10.43	**<0.001**
Direct Bilirubin (mg/dL)	0.10 ± 0.04	3.55 ± 4.87	**<0.001**
Albumin (g/dL)	4.28 ± 0.44	3.63 ± 0.70	**<0.001**
Total Protein (g/dL)	7.23 ± 0.82	6.56 ± 0.99	**0.002**
GGT (U/L)	22.00 ± 4.52	87.60 ± 12.55	**<0.001**
ALP (U/L)	255.32 ± 20.59	371.47 ± 34.15	**0.005**
LDH (U/L)	257.41 ± 15.54	294.06 ± 16.82	0.185

Mean ± SD: Mean ± Standard Deviation; BMI: Body Mass Index; MUAC: Mid-Upper Arm Circumference; WBC: White Blood Cell count; RBC: Red Blood Cell count; PCT: Plateletcrit; MCV: Mean Corpuscular Volume; PDW: Platelet Distribution Width; RDW: Red Cell Distribution Width; AST: Aspartate Aminotransferase; ALT: Alanine Aminotransferase; GGT: Gamma-Glutamil Transferaz; ALP: Alkaline Phosphatase; LDH: Lactate Dehydrogenase. Statistical analysis methods: * Chi-square test (Cross-tabulation); ** Independent Student’s *t*-test. Statistically significant *p*-values are indicated in bold.

**Table 2:** Comparative Analysis of Hemodynamic and Echocardiographic Parameters Between the Groups.

Parameters	Cirrhosis Group (*n* = 75) Mean ± SD	Control Group (*n* = 61) Mean ± SD	*p*-Value
Systolic BP (mmHg)	103.52 ± 16.47	107.06 ± 13.98	0.183
Diastolic BP (mmHg)	62.79 ± 12.36	63.20 ± 9.70	0.836
HR (beats/min)	116.30 ± 25.33	104.36 ± 25.53	**0.008**
Oxygen Saturation (%)	97.30 ± 2.38	98.62 ± 1.24	**<0.001**
LVIDd (mm)	35.89 ± 9.57	34.96 ± 9.00	0.567
LVIDs (mm)	20.98 ± 6.06	21.32 ± 5.79	0.743
IVSd (mm)	6.24 ± 1.83	6.10 ± 1.65	0.638
IVSs (mm)	9.22 ± 2.58	8.43 ± 1.96	**0.044**
LVPWd (mm)	6.65 ± 1.72	6.71 ± 1.83	0.860
LVPWs (mm)	10.72 ± 2.26	10.32 ± 2.47	0.322
Ejection Fraction (EF, %)	73.40 ± 5.66	70.87 ± 5.30	**0.012**
Fractional Shortening (FS, %)	41.74 ± 4.90	39.55 ± 4.68	**0.009**
Aortic Annulus (mm)	13.23 ± 4.31	13.90 ± 4.34	0.371
Ascending Aorta (mm)	15.38 ± 4.70	16.33 ± 4.70	0.244
Aortic VTI (cm)	23.86 ± 5.91	19.66 ± 4.51	**<0.001**
Pulmonary Annulus (mm)	14.50 ± 4.59	15.23 ± 4.72	0.364
Main Pulmonary Artery (mm)	15.78 ± 4.80	17.02 ± 4.71	0.131
Pulmonary VTI (cm)	24.18 ± 5.03	20.53 ± 4.22	**<0.001**
Mitral E (m/s)	0.98 ± 0.19	0.84 ± 0.13	**<0.001**
Mitral A (m/s)	0.65 ± 0.15	0.62 ± 0.15	0.165
Mitral E/A Ratio	1.38 ± 0.36	1.58 ± 0.46	**0.010**
Mitral a′ (m/s)	0.09 ± 0.02	0.07 ± 0.02	**0.002**
Mitral e′ (m/s)	0.12 ± 0.03	0.12 ± 0.02	0.156
Mitral e′/a′	1.38 ± 0.36	1.58 ± 0.47	**0.006**
Mitral E/e′	8.65 ± 2.71	7.05 ± 2.72	**0.001**
Mitral DT (ms)	103.12 ± 28.63	86.70 ± 25.89	**0.001**
IVRT (ms)	54.37 ± 13.19	44.45 ± 11.15	**<0.001**
IVCT (ms)	49.21 ± 13.07	43.39 ± 11.54	**0.007**
Ejection Time (ET, ms)	257.13 ± 37.79	269.95 ± 47.57	0.082
Myocardial Performance Index (MPI)	0.41 ± 0.09	0.33 ± 0.07	**<0.001**
TAPSE (mm)	19.18 ± 4.20	17.17 ± 3.96	**0.005**
Tricuspid E (m/s)	0.84 ± 0.15	0.71 ± 0.11	**<0.001**
Tricuspid A (m/s)	0.62 ± 0.13	0.54 ± 0.11	**<0.001**
RVESA (cm^2^)	39.19 ± 10.26	41.61 ± 10.52	0.178
RVEDA (cm^2^)	53.48 ± 12.52	53.49 ± 13.42	0.998
FAC (%)	52.66 ± 9.15	49.23 ± 8.07	**0.023**
Left Atrial Area (cm^2^)	9.11 ± 4.62	8.44 ± 3.52	0.353

SD: Standard Deviation; BP: Blood Pressure; HR: Heart Rate; LVIDd: Left Ventricular Internal Diameter in Diastole; LVIDs: Left Ventricular Internal Diameter in Systole; IVSd: Interventricular Septal Thickness in Diastole; IVSs: Interventricular Septal Thickness in Systole; LVPWd: Left Ventricular Posterior Wall Thickness in Diastole; LVPWs: Left Ventricular Posterior Wall Thickness in Systole; EF: Ejection Fraction; FS: Fractional Shortening; VTI: Velocity-Time Integral; Mitral E: Peak Early Diastolic Mitral Inflow Velocity; Mitral A: Peak Late Diastolic Mitral Inflow Velocity; Mitral e′: Early Diastolic Mitral Annular Velocity; Mitral a′: Late Diastolic Mitral Annular Velocity; DT: Deceleration Time; IVRT: Isovolumetric Relaxation Time; IVCT: Isovolumetric Contraction Time; ET: Ejection Time; MPI: Myocardial Performance Index (Tei Index); TAPSE: Tricuspid Annular Plane Systolic Excursion; RVESA: Right Ventricular End-Systolic Area; RVEDA: Right Ventricular End-Diastolic Area; FAC: Fractional Area Change; ms: milliseconds. Note: Data are presented as Mean ± Standard Deviation. Independent Student’s *t*-test was used for statistical comparison. Statistically significant *p*-values are indicated in bold.

**Table 3:** Evaluation of Cardiac Findings According to Compensation Status in Liver Cirrhosis.

Parameters	Control Mean ± SD	Compensated Cirrhosis Mean ± SD	Decompensated Cirrhosis Mean ± SD	*p*-Value *
Systolic BP (mmHg)	107.06 ± 13.90	104.09 ± 17.48	102.70 ± 15.15	0.397
Diastolic BP (mmHg)	63.20 ± 9.70	63.46 ± 13.29	61.83 ± 11.03	0.814
HR (beats/min)	104.36 ± 25.53 ^β^	115.41 ± 28.13	117.56 ± 21.07 ^α^	**0.027**
Oxygen Saturation (%)	98.62 ± 1.24 ^β^	97.58 ± 1.27 ^α^	96.90 ± 1.24 ^α^	**<0.001**
LVIDd (mm)	34.96 ± 9.00	34.10 ± 10.04	38.57 ± 8.27	0.105
LVIDs (mm)	21.32 ± 5.79	19.89 ± 6.31	22.62 ± 5.36	0.142
IVSd (mm)	6.10 ± 1.66	6.41 ± 1.85	5.99 ± 1.79	0.527
IVSs (mm)	8.43 ± 1.96	9.37 ± 2.76	9.00 ± 2.31	0.118
LVPWd (mm)	6.71 ± 1.83	6.46 ± 1.76	6.94 ± 1.65	0.514
LVPWs (mm)	10.31 ± 2.47	10.71 ± 2.53	10.73 ± 1.83	0.614
Ejection Fraction (EF, %)	70.86 ± 5.83 ^β^	73.66 ± 5.86 ^α^	73.00 ± 5.3 ^α^	**0.037**
Fractional Shortening (FS, %)	39.55 ± 4.68 ^β^	41.82 ± 5.22 ^α^	41.63 ± 4.46 ^α^	**0.034**
Aortic Annulus (mm)	13.17 ± 3.71	13.27 ± 4.71	13.17 ± 3.71	0.668
Ascending Aorta (mm)	16.33 ± 4.70	15.60 ± 5.16	15.06 ± 3.98	0.452
Aortic VTI (cm)	19.66 ± 4.51 ^β^	23.17 ± 5.62 ^α^	24.89 ± 6.26 ^α^	**<0.001**
Pulmonary Annulus (mm)	15.23 ± 4.72	14.38 ± 4.96	14.67 ± 4.05	0.640
Main Pulmonary Artery (mm)	17.02 ± 4.71	15.81 ± 5.07	15.72 ± 4.46	0.321
Pulmonary VTI (cm)	20.53 ± 4.22 ^β^	23.21 ± 4.58 ^α^	25.63 ± 5.40 ^α^	**<0.001**
Mitral E (m/s)	0.84 ± 0.13 ^β^	0.92 ± 0.16 ^α^	1.06 ± 0.21 ^α^	**<0.001**
Mitral A (m/s)	0.62 ± 0.14	0.62 ± 0.13	0.69 ± 0.16	0.054
Mitral E/A Oranı	1.58 ± 0,46 ^β^	1.36 ± 0.39 ^α^	1.42 ± 0.34	0.019
Mitral a′ (m/s)	0.07 ± 0.02 ^β^	0.09 ± 0.03 ^α^	0.09 ± 0.02	0.015
Mitral e′ (m/s)	1.58 ± 0.46 ^β^	1.35 ± 0.38 ^α^	1.42 ± 0.34	**0.019**
Mitral e′/a′ Oranı	1.58 ± 0.47 ^β^	1.35 ± 0.39 ^α^	1.42 ± 0.34	0.020
Mitral E/e′ Oranı	7.05 ± 2.7 ^β^	8.38 ± 2.78 ^α^	9.05 ± 2.6 ^α^	0.002
Mitral DT (ms)	86.70 ± 25.89 ^β^	101.11 ± 27.67 ^α^	106.13 ± 30.23 ^α^	**0.002**
IVRT (ms)	44.45 ± 11.15 ^β^	55.51 ± 13.78 ^α^	52.66 ± 12.30 ^α^	**<0.001**
IVCT (ms)	43.39 ± 11.54 ^β^	50.13 ± 14.06 ^α^	47.83 ± 11.52	**0.021**
Ejection Time (ET, ms)	269.95 ± 47.56	258.22 ± 32.54	255.50 ± 45.09	0.214
MPI	0.33 ± 0.07 ^β^	0.42 ± 0.10 ^α^	0.39 ± 0.08 ^α^	**<0.001**
TAPSE (mm)	17.17 ± 3.96 ^β^	18.52 ± 4.47	20.18 ± 3.58 ^α^	**0.005**
Tricuspid E (m/s)	0.71 ± 0.11 ^β^	0.81 ± 0.13 ^α^	0.86 ± 0.16 ^α^	**<0.001**
Tricuspid A (m/s)	0.54 ± 0.11 ^β^	0.61 ± 0.14 ^α^	0.63 ± 0.11 ^α^	**0.001**
RVESA (cm^2^)	41.62 ± 10.52	38.76 ± 10.06	39.84 ± 10.67	0.367
RVEDA (cm^2^)	53.49 ± 13.42	52.87 ± 13.75	54.42 ± 10.56	0.880
FAC (%)	49.22 ± 8.07	52.46 ± 9.42	52.96 ± 8.87	0.075
Left Atrial Area (cm^2^)	8.44 ± 3.52	8.90 ± 4.81	8.44 ± 3.52	0.565

SD: Standard Deviation; BP: Blood Pressure; HR: Heart Rate; LVIDd: Left Ventricular Internal Diameter in Diastole; LVIDs: Left Ventricular Internal Diameter in Systole; IVSd: Interventricular Septal Thickness in Diastole; IVSs: Interventricular Septal Thickness in Systole; LVPWd: Left Ventricular Posterior Wall Thickness in Diastole; LVPWs: Left Ventricular Posterior Wall Thickness in Systole; EF: Ejection Fraction; FS: Fractional Shortening; VTI: Velocity-Time Integral; Mitral E: Peak Early Diastolic Mitral Inflow Velocity; Mitral A: Peak Late Diastolic Mitral Inflow Velocity; Mitral e′: Early Diastolic Mitral Annular Velocity; Mitral a′: Late Diastolic Mitral Annular Velocity; DT: Deceleration Time; IVRT: Isovolumetric Relaxation Time; IVCT: Isovolumetric Contraction Time; ET: Ejection Time; MPI: Myocardial Performance Index (Tei Index); TAPSE: Tricuspid Annular Plane Systolic Excursion; RVESA: Right Ventricular End-Systolic Area; RVEDA: Right Ventricular End-Diastolic Area; FAC: Fractional Area Change; ms: milliseconds. Statistics: One-Way ANOVA. Data are presented as Mean ± Standard Deviation. Statistically significant *p*-values are indicated in bold. * There are statistically significant differences between α and β. α Indicates a statistically significant difference in the respective cirrhosis group (Compensated or Decompensated) compared to the Healthy Control group (Post hoc Tukey/Dunnett, *p* < 0.05). β Indicates a statistically significant difference in the Control group compared to the Compensated cirrhosis group (Post hoc Tukey/Dunnett, *p* < 0.05).

**Table 4:** Correlation Analysis Between Cardiovascular Parameters, Liver Prognostic Scores, and Non-Invasive Fibrosis Indices.

Cardiovascular Parameters	Statistics	MELD	PELD	FIB-4	AAR	APRI
HR (beats/min)	Pearson r	0.123	0.408	−0.087	0.24	0.221
	*p*-value	0.704	0.002 *	0.439	0.03	0.046
Oxygen Saturation (%)	Pearson r	0.009	0.131	0.032	0.019	−0.237
	*p*-value	0.978	0.341	0.774	0.868	0.032
Aortic VTI (cm)	Pearson r	−0.235	−0.102	0.261	−0.209	0.059
	*p*-value	0.461	0.451	0.016	0.056	0.594
EF (%)	Pearson r	−0.210	0.3	−0.073	0.045	0.292
	*p*-value	0.511	0.023	0.507	0.687	0.007
FS (%)	Pearson r	−0.157	0.248	−0.015	−0.025	0.269
	*p*-value	0.625	0.063	0.893	0.824	0.013
Pulmonary VTI (cm)	Pearson r	−0.098	0.034	0.421	−0.079	0.25
	*p*-value	0.762	0.8	<0.0001 **	0.476	0.022
Mitral E/A	Pearson r	−0.019	−0.310	0.112	−0.202	−0.046
	*p*-value	0.953	0.019	0.311	0.065	0.681
Mitral E/e′	Pearson r	0.256	0.433	−0.152	0.361	0.166
	*p*-value	0.421	0.001 *	0.168	0.001	0.132
Annular Mitral e′/a′	Pearson r	−0.019	−0.300	0.08	−0.196	−0.083
	*p*-value	0.953	0.023	0.467	0.074	0.455
Mitral DT (ms)	Pearson r	0.003	−0.038	0.167	−0.107	0.134
	*p*-value	0.992	0.781	0.128	0.335	0.225
IVRT (ms)	Pearson r	−0.376	−0.009	0.041	−0.104	−0.021
	*p*-value	0.228	0.944	0.711	0.347	0.852
IVCT (ms)	Pearson r	−0.523	−0.177	−0.057	−0.136	−0.162
	*p*-value	0.081	0.189	0.604	0.216	0.142
MPI	Pearson r	−0.305	−0.016	0.02	−0.083	0.031
	*p*-value	0.335	0.904	0.854	0.452	0.781
Tricuspid E (m/s)	Pearson r	−0.017	0.264	0.209	0.045	0.343
	*p*-value	0.959	0.048	0.057	0.681	0.001
Tricuspid A (m/s)	Pearson r	0.667	0.297	0.126	0.117	0.208
	*p*-value	0.018	0.025	0.254	0.289	0.058
TAPSE (mm)	Pearson r	−0.424	−0.174	0.333	−0.255	−0.102
	*p*-value	0.169	0.196	0.002	0.019	0.357

MELD: Model for End-Stage Liver Disease; PELD: Pediatric End-Stage Liver Disease; FIB-4: Fibrosis-4 Index; AAR: AST/ALT Ratio; APRI: AST to Platelet Ratio Index; HR: Heart Rate; EF: Ejection Fraction; FS: Fractional Shortening; VTI: Velocity-Time Integral; DT: Deceleration Time; IVRT: Isovolumetric Relaxation Time; IVCT: Isovolumetric Contraction Time; MPI: Myocardial Performance Index; TAPSE: Tricuspid Annular Plane Systolic Excursion; ms: milliseconds Statistical analysis was performed using Pearson correlation analysis. To control for the inflation of Type I error rate across multiple simultaneous tests, a Bonferroni multiplicity correction was applied. Statistically robust correlations surviving the family-wise column adjustment (*p* < 0.003125) are indicated with double asterisks (**), while those achieving the conservative global significance threshold (*p* < 0.000625) are highlighted with a single asterisk (*). Non-adjusted exploratory *p*-values between 0.003125 and 0.05 are presented strictly for descriptive trend visibility and are not interpreted as independent clinical discoveries. Statistically significant *p*-values are indicated in bold.

**Table 5:** Evaluation of Cardiac and Hemodynamic Parameters in Predicting Decompensated Cirrhosis Using ROC Curve Analysis.

Parameters	Cut-Off Value	AUC	95% CI	Sensitivity (%)	Specificity (%)	PPV (%)	NPV (%)	*p*-Value
Mitral E (m/s)	>1.08	0.758	0.677–0.827	53.33	91.51	64.00	87.40	**<0.001**
Pulmonary VTI (cm)	>23.00	0.727	0.644–0.800	76.67	65.09	38.30	90.80	**<0.001**
Tricuspid E (m/s)	>0.81	0.691	0.606–0.768	63.33	72.64	39.60	87.50	**0.001**
Aortic VTI (cm)	>23.40	0.679	0.594–0.757	60.00	74.53	40.00	86.80	**0.002**
Tricuspid A (m/s)	>0.63	0.669	0.583–0.747	56.67	72.64	40.50	86.20	**0.004**
TAPSE (mm)	>20.40	0.668	0.582–0.747	50.00	76.42	37.50	84.40	**0.004**
Mitral E/e′	>6.14	0.654	0.568–0.734	100.00	29.25	28.60	100.00	**0.009**
Oxygen Saturation (%)	≤98.00	0.622	0.534–0.705	83.30	41.35	29.10	89.20	**0.026**

AUC: Area Under the Curve; PPV: Positive Predictive Value; NPV: Negative Predictive Value; VTI: Velocity-Time Integral; TAPSE: Tricuspid Annular Plane Systolic Excursion. Statistical analysis: ROC (Receiver Operating Characteristic) curve analysis was utilized. Statistically significant *p*-values are indicated in bold.

## Data Availability

The original contributions presented in this study are included in the article. Further inquiries can be directed to the corresponding author.

## Abbreviations

PELD	Pediatric End-Stage Liver Disease
MELD	Model for End-Stage Liver Disease
FİB-4 Score	Fibrosis-4 Index
APRI	AST to Platelet Ratio Index
AAR	AST/ALT Ratio
PW	Pulse Wave
CW	Continuous-wave
TDI	Tissue Doppler Imaging
SD	Standard Deviation
BP	Blood Pressure
LVIDd	Left Ventricular Internal Diameter in Diastole
LVIDs	Left Ventricular Internal Diameter in Systole
IVSd	Interventricular Septal Thickness in Diastole
IVSs	Interventricular Septal Thickness in Systole
LVPWd	Left Ventricular Posterior Wall Thickness in Diastole
LVPWs	Left Ventricular Posterior Wall Thickness in Systole
EF	Ejection Fraction
FS	Fractional Shortening
VTI	Velocity-Time Integral
Mitral E	Peak Early Diastolic Mitral Inflow Velocity
Mitral A	Peak Late Diastolic Mitral Inflow Velocity
Mitral e′	Early Diastolic Mitral Annular Velocity
Mitral a′	Late Diastolic Mitral Annular Velocity
DT	Deceleration Time
IVRT	Isovolumetric Relaxation Time
IVCT	Isovolumetric Contraction Time
ET	Ejection Time
MPI	Myocardial Performance Index (Tei Index)
TAPSE	Tricuspid Annular Plane Systolic Excursion
RVESA	Right Ventricular End-Systolic Area
RVEDA	Right Ventricular End-Diastolic Area
FAC	Fractional Area Change
ms	milliseconds
CCM	Cirrhotic cardiomyopathy
BMI	Body Mass Index
MUAC	Mid-Upper Arm Circumference
WBC	White Blood Cell count
RBC	Red Blood Cell count
PCT	Plateletcrit
MCV	Mean Corpuscular Volume
PDW	Platelet Distribution Width
RDW	Red Cell Distribution Width
AST	Aspartate Aminotransferase
ALT	Alanine Aminotransferase
GGT	Gamma-Glutamyl Transferase
ALP	Alkaline Phosphatase
LDH	Lactate Dehydrogenase
AUC	Area Under the Curve
PPV	Positive Predictive Value
NPV	Negative Predictive Value

## References

[1] Izzy M., VanWagner L.B., Lin G., Altieri M., Findlay J.Y., Oh J.K., Watt K.D., Lee S.S., Cirrhotic Cardiomyopathy Consortium (2020). Redefining Cirrhotic Cardiomyopathy for the Modern Era. Hepatology.

[2] Yoon K.T., Liu H., Lee S.S. (2020). Cirrhotic Cardiomyopathy. Curr. Gastroenterol. Rep..

[3] Møller S., Henriksen J.H. (2008). Cardiovascular complications of cirrhosis. Gut.

[4] Wong F. (2009). Cirrhotic cardiomyopathy. Hepatol. Int..

[5] Almeida F., Sousa A. (2024). Cirrhotic cardiomyopathy: Pathogenesis, clinical features, diagnosis, treatment and prognosis. Rev. Port. Cardiol..

[6] Møller S., Henriksen J.H. (2010). Cirrhotic cardiomyopathy. J. Hepatol..

[7] Radu T., Iacob S., Gheorghe L. (2025). Cirrhotic Cardiomyopathy: Mechanisms, Diagnostic Tools and Therapeutic Options. J. Gastrointest. Liver Dis..

[8] Karki N., Kc S., Sharma D., Jaisi B., Khadka S. (2019). Cardiac Dysfunction in Patients with Liver Cirrhosis. J. Nepal Health Res. Counc..

[9] Fede G., Privitera G., Tomaselli T., Spadaro L., Purrello F. (2015). Cardiovascular dysfunction in patients with liver cirrhosis. Ann. Gastroenterol..

[10] Rabadia J.P., Thite V.S., Desai B.K., Bera R.G., Patel S. (2024). Cardiovascular system, its functions and disorders. Cardioprotective Plants.

[11] Gananandan K., Wiese S., Møller S., Mookerjee R.P. (2024). Cardiac dysfunction in patients with cirrhosis and acute decompensation. Liver Int..

[12] Dadhich S., Goswami A., Jain V.K., Gahlot A., Kulamarva G., Bhargava N. (2014). Cardiac dysfunction in cirrhotic portal hypertension with or without ascites. Ann. Gastroenterol..

[13] Niculae A.Ș., Căinap S.S., Grama A., Pop T.L. (2024). Pediatric cirrhotic cardiomyopathy: Literature review and effect size estimations of selected parameters. Eur. J. Pediatr..

[14] Wu Z.P., Wang Y.F., Shi F.W., Cao W.H., Sun J., Yang L., Ding F.P., Hu C.X., Kang W.W., Han J. (2025). Predictive models and clinical manifestations of intrapulmonary vascular dilatation and hepatopulmonary syndrome in patients with cirrhosis: Prospective comparative study. World J. Gastroenterol..

[15] Nagueh S.F., Middleton K.J., Kopelen H.A., Zoghbi W.A., Quiñones M.A. (1997). Doppler tissue imaging: A noninvasive technique for evaluation of left ventricular relaxation and estimation of filling pressures. J. Am. Coll. Cardiol..

[16] Lyu H., Ye Y., Wang B. (2024). FIB-4 and APRI scores for progressive liver fibrosis diagnosis in children with biliary atresia. Front. Pediatr..

[17] Chinese Society of Hepatology, Chinese Medical Association (2019). Chinese guidelines on the management of liver cirrhosis. Zhonghua Gan Zang Bing Za Zhi.

[18] Brunt E.M. (2016). Nonalcoholic Fatty Liver Disease: Pros and Cons of Histologic Systems of Evaluation. Int. J. Mol. Sci..

[19] Fibrosis-4 (FIB-4) Calculator. https://www.hepatitisc.uw.edu/page/clinical-calculators/fib-4.

[20] AST to Platelet Ratio Index (APRI) Calculator. https://www.hepatitisc.uw.edu/page/clinical-calculators/apri.

[21] Botros M., Sikaris K.A. (2013). The de ritis ratio: The test of time. Clin. Biochem. Rev..

[22] MELD Na (UNOS/OPTN). https://www.mdcalc.com/calc/78/meld-score-model-end-stage-liver-disease-12-older.

[23] PELD Score (Pediatric End-Stage Liver Disease) (Younger than 12). https://www.mdcalc.com/calc/87/peld-score-pediatric-end-stage-liver-disease-younger-12.

[24] Polat T.B., Urganci N., Yalcin Y., Akdeniz C., Zeybek C., Erdem A., Celebi A. (2006). Evaluation of cardiac function by tissue Doppler imaging in children with chronic hepatitis. J. Pediatr. Gastroenterol. Nutr..

[25] Rabie R.N., Cazzaniga M., Salerno F., Wong F. (2009). The use of E/A ratio as a predictor of outcome in cirrhotic patients treated with transjugular intrahepatic portosystemic shunt. Am. J. Gastroenterol..

[26] Somani P.O., Contractor Q., Chaurasia A.S., Rathi P.M. (2014). Diastolic dysfunction characterizes cirrhotic cardiomyopathy. Indian Heart J..

[27] Çelik E., Karakurt C., Çelik S.F., Selimoğlu A., Varol F.İ. (2017). Evaluation of cardiac functions in cirrhotic children using tissue Doppler imaging. Akad. Gastroenteroloji Derg..

[28] Fattouh A.M., El-Shabrawi M.H., Mahmoud E.H., Ahmed W.O. (2016). Evaluation of cardiac functions of cirrhotic children using serum brain natriuretic peptide and tissue Doppler imaging. Ann. Pediatr. Cardiol..

[29] Meric M., Yesildag O., Yuksel S., Soylu K., Arslandag M., Dursun I., Zengin H., Koprulu D., Yilmaz O. (2014). Tissue doppler myocardial performance index in patients with heart failure and its relationship with haemodynamic parameters. Int. J. Cardiovasc. Imaging.

[30] López-Candales A., Menendez F.L., Shah S.A., Friedrich A. (2014). Measures of right ventricular systolic function in end stage liver disease patients awaiting transplant. Int. J. Cardiol..

[31] Zhang K., Braun A., von Koeckritz F., Schmuck R.B., Teegen E.M., Cuspidi C., Heinzel F., Pieske B., Tadic M. (2019). Right Heart Remodeling in Patients with End-Stage Alcoholic Liver Cirrhosis: Speckle Tracking Point of View. J. Clin. Med..

[32] Kim M.Y., Baik S.K., Kim M.Y., Baik S.K. (2009). Hyperdynamic Circulation in Patients with Liver Cirrhosis and Portal Hypertension. Korean J. Gastroenterol. = Taehan Sohwagi Hakhoe Chi.

[33] Maryam A., Amir N., Mahmood H., Mohsen D., Hamid A., Gholamreza K., Nima M., Leila S. (2025). Cirrhotic cardiomyopathy in children: A comprehensive assessment using ECG, echocardiography, and NT-Pro-BNP. Clin. Res. Hepatol. Gastroenterol..

[34] Bansal N., Mahgerefteh J., Lamour J.M., Kogan-Liberman D., Ovchinsky M., Ganzburg K., Choueiter N. (2025). Evaluation of Cardiac Function in Children Undergoing Liver Transplantation. Pediatr. Cardiol..

[35] Nagi S.A., Zakaria H., Elkhadry S.W., Hamed W., Gaballa N., Elkholy S. (2023). APRI and FIB-4 indices as diagnostic noninvasive scores for prediction of severe fibrosis in patients with biliary atresia. Clin. Exp. Hepatol..

[36] Junge N., Junge C., Schröder J., Pfister E.D., Leiskau C., Hohmann D., Beerbaum P., Baumann U. (2018). Pediatric cirrhotic cardiomyopathy: Impact on liver transplant outcomes. Liver Transplant..

